# The written history of plant phenology: shaping primary sources for secondary publications

**DOI:** 10.1007/s00114-023-01861-w

**Published:** 2023-07-06

**Authors:** Jari Holopainen, Samuli Helama, Henry Väre

**Affiliations:** 1 Natural Resources Institute Finland, Helsinki, Finland; 2 Natural Resources Institute Finland, Rovaniemi, Finland; 3 Finnish Museum of Natural History, Botanical Museum, University of Helsinki, Helsinki, Finland

**Keywords:** Phenology, Historical data, Data comparison, Citizen science, Community involvement

## Abstract

Phenological research establishes the science of nature's natural calendar. This research, the monitoring and analysis of seasonal rhythms of plants and animals, is commonly based on citizen science data. Such data may be digitized from primary sources provided by the citizen scientist’s original phenological diaries. Secondary data sources are formed by historical publications (for example, yearbooks and climate bulletins). While primary data has the advantage of first-hand notetaking, its digitization may, in practice, be time-consuming. Contrastingly, secondary data can contain well-organized typesetting, making digitization less labour-intensive. However, secondary data can be reshaped by the motivations of the historical actors who were collating the data. This study compared data from 1876–1894 gathered originally by citizen scientists (primary data) and the secondary data founded upon the previous primary data, later published by the Finnish Society of Sciences and Letters as a series of phenological yearbooks. In the secondary data, the recorded numbers of taxa and their phenological stages appeared to be fewer and phenological events standardized, with an increased prevalence of agricultural phenology (at the cost of autumn phenology). Moreover, it seems the secondary data had been screened for potential outliers. While secondary sources may provide current phenologists with coherent sets of relevant data, future users must be aware of potential data reshaping resulting from the preferences of historical actors. These actors may weigh and limit the original observations according to their own criteria and preferences.

**Supplementary Information:**

The online version contains supplementary material available at 10.1007/s00114-023-01861-w.

## Introduction

Phenological observations provide data on seasonal changes in natural processes. The changes in the annual cycles of plants, often described by phenological data, are closely linked to climatic factors, especially temperature variability. Phenological data constitute primary evidence of plants' responses to changing seasons and can be used to analyse ongoing climatic trends in the context of late Holocene vegetative history. Thus, phenological observations are especially useful in global change research (Menzel 2002). These records demonstrate the average advance of spring and summer in accordance with instrumentally observed warming (Menzel et al. 2006, 2020; Helama et al. 2020; Kalvāne and Kalvāns 2021). Furthermore, they help identify alterations in plant development through phenological stages and their climatic drivers under enhanced warming and variable moisture conditions (Cook and Wolkovich 2016). Moreover, these data contribute to a wider knowledge of historical ecology (Sheail 1980), with large amounts of documentary evidence of past ecological conditions. Commonly, phenological data originate from citizen science contributions as the collection of the data has traditionally been, to a considerable degree, carried out by volunteers enthused by the natural world (Mayer 2010; Posthumus and Crimmins 2011; Kobori et al. 2016).

A significant concern about citizen science is the quality and reliability of data to be used for scientific analyses. Regarding this, Fuccillo et al. (2015) evaluated the accuracy of circa 11000 plant phenology observations made by volunteers receiving several hours of formal training by comparing this citizen science data with observations made by a professional ecologist over one field season. Both types of observations were made following protocols designed by the USA National Phenology Network. Overall, the results of Fuccillo et al. (2015) suggested that the citizen scientists of their study provided reliable observations when following explicit, standardized protocols. Additionally, Bison et al. (2019) analysed data on annual tree phenology collected by volunteers from the western European Alps over twelve years. They found decadal-scale shifts in budburst date consistent with results from other studies, concluding that citizen science data can significantly contribute to climate change studies. An essential approach to understanding and improving the quality of citizen science data is the detection of statistical outliers. Li et al. (2020) compared the use of several such methods for data (1987–2016) from a plant phenology network in Alberta (Canada). Removal of outliers was found to be a simple and effective method to improve the reliability of their citizen science phenology observations. Despite these encouraging results, we also note that citizen science data may be prone to problems concerning temporal coverage of observations (Courter et al. 2013), representativeness of site habitats and observed taxa (Brereton et al. 2014), and taxonomic misidentification (Vantieghem et al. 2017).

In addition to using recent phenological ground-based observations, there has been an increased effort to utilize previously unused secondary sources of historical phenological data (Burris et al. 2019; Petrauski et al. 2020; Fitchett and Raik 2021). Such data from historical collections and sources provide information on ongoing phenological changes with a long-term perspective, i.e., the natural variability with which the recent phenological trends can be meaningfully assessed. Recently, Burris et al. (2019) extracted weather and climate data from a historical plantation document in Virginia (USA), including direct weather observations and information on various cultivars, and compared them to more current data (1943–2017) of final spring freeze events in their sites. Fitchett and Raik (2021) analysed the peak flowering dates of an invasive tree species *Jacaranda mimosifolia*, reported in local newspapers in the Gauteng City-Region in South Africa. They found an advance of 2.1 days per decade (1927–2019) in the flowering date over the full phenological record. Petrauski et al. (2020) discussed the development of their recent project, which uses historical (1890–2015) phenology data from citizens in West Virginia (USA). Their data were obtained from archives of local naturalists' clubs and personal records of citizen scientists, as well as herbarium samples (pressed flower specimens) and dated photographs.

In Finland, the collection of plant phenological data has primarily been done through citizen science, with the support of early naturalists and academic phenologists. These ground-based observations are made by visually observing the seasonal development of the plants and their phenological stages. Initially, the activity is known to have started when Johan Leche, the professor of medicine at the Royal Academy of Turku, started his observations in southwest Finland in 1750 (Moberg 1857; Leche and Hjelt 1889; Häkkinen 1999; Holopainen 2004; Holopainen et al. 2012). The need for large phenological datasets was previously declared by Carl von Linné (Dahl and Langvall 2008). In the following years, collecting phenological observations in Finland was perpetuated and organized by the personnel at the Royal Academy of Turku, the Pro Natura Society, and the Finnish Economic Society, inviting the educated class of society to participate in the activity. The Finnish Society of Sciences and Letters (*Suomen Tiedeseura*) was later established in 1838 and took over the collections of earlier phenological observations, including the responsibility for any new data collection from 1846 onwards (Elfving 1938; Elfving and Mickwitz 1988). Phenological data collection continued with sustained persistence, with hundreds of volunteers contributing to the national effort. Notebooks returned to the Society were collated and made publicly available through the 19th and early part of the twentieth century. The initiator of this undertaking was Professor Adolf Moberg, who compiled many original 18th and 19^th^-century observations into what became a series of works published in Swedish. These works presented the selected phenological data from Finland in a well-organized format: as tabular lists of various natural events and their seasonal timing.

Previously, Holopainen et al. (2018) presented and illustrated the historical plant phenological data from the monographs of Moberg (1857, 1860, 1885a, 1894a). This data compiled 44487 observations from 450 different plant taxa for 15 different phenological stages from 193 sites across Finland and covered the early years of phenological data collection from 1750 to 1875 (Holopainen et al. 2018). More recently, the work was continued with a new set of plant phenological data digitized from historical publications covering 1876–1965 (with a gap for 1956–1960). Originally published by the Finnish Society of Sciences and Letters, the combined dataset of plant phenological observations (1750–1965) totalled 265478 observations from 985 taxa for 16 different phenological stages made at 371 locations across the country (Holopainen et al. 2023). The collection of phenological observations has continued since it was first organised by the Finnish Society of Sciences and Letters and the Museum of Natural History, University of Finland (Elfving and Mickwitz 1988; Holopainen et al. 2012). The resulting dataset of this continued collection collates observations, of which partial compositions (frequently focusing on Betula time series) have been previously used for estimating the changes in phenological stages and their relatedness to past climates from the eighteenth century to present day (Lappalainen and Heikinheimo 1992; Heikinheimo and Lappalainen 1997; Häkkinen et al. 1995, 1998; Linkosalo et al. 1996, 2000, 2008, 2009; Linkosalo 2000; Holopainen et al. 2006, 2012, 2013, 2018, 2023; Hari et al. 2017). These studies demonstrate the magnitude of citizen science involvement in Finnish phenological research. Compared to analyses of current data, however, the use of phenological data from historical publications has become more complex. This complexity comes from how the data is presented in historical publications (e.g., yearbooks, monthly or weekly climate reviews and/or bulletins; Koch et al. 2007) and how it may differ in composition from those initially made by the volunteers.

Here, we aim to investigate how the properties of data from historical publications differ from the original phenological diaries kept by volunteers in the context of Finnish phenological data. A new dataset of plant phenological observations was digitized from printed booklets, which had been sent to volunteers by the Finnish Society of Sciences and Letters as formal diaries during 1876–1894. Following established terminology (Hox and Boeije 2005), these research materials constitute the primary data source of our study. The data from these booklets, returned to and archived by the Society, were compared with those the Society had made available through their yearly publications. The published versions of the material are our secondary data sources in this study (Hox and Boeije 2005). While it is known that not all of the primary data was included in the historical publications (Holopainen et al. 2023), we used a set of questions to address specific targets in our analysis: (1) how much were the primary data withheld from the secondary data; (2) what percentage of species and phenological stages were withheld from the secondary data; (3) are the distributions of the species, phenological stages and sites weighted differently in the primary and secondary data, for example, has the proportion of observations from natural and arable ecosystems, or the distribution of sites across the country changed, particularly when part of the data was included, or some of the data excluded from secondary sources. Moreover, the method of Li et al. (2020) was used to estimate potential outliers in the data by a z-score statistic. Performing this estimation for both datasets makes it possible to evaluate (4) whether the observations suspected as outliers had been excessively withheld from publications. These questions are essential for understanding the use of secondary data, which may not represent the spectrum of data once observed across sites and ecosystems, in analysing ecosystem dynamics and their responses to a changing environment from local to global scales.

## Materials and methods

### Primary data

Formal phenological diaries or notebooks from 1876–1894 were investigated. The notebooks were photographed at the National Archives of Finland. The information available from the photographs was manually typed into an electronic format and saved in Microsoft Excel. Later, the data were screened for obvious mistakes and crosschecked by team members. Each phenological observation was characterized by the plant species, phenological stage (e.g., budburst, flowering), year, month, day of the month, as well as the site name and geographical coordinates. In the case of agrophenological observations, the phenological stages were related to human activity (e.g., sowing of rye). Some events were identifiable to the phenological substage, for example, the beginning of the rowan flowering. These data were digitized to substitute the plant phenological data published in the historical yearbooks entitled ‘*Öfversigt af Finska Vetenskaps-Societetens förhandlingar*’ (Moberg 1878a, b, 1879, 1880, 1881, 1882, 1883, 1884, 1885b, 1886, 1887, 1888, 1889, 1890, 1891, 1892, 1893, 1894b; Kihlman 1895), to compare the conformability of the primary, ‘raw’ data with those available from historical publications. There was no reason to concentrate on this period (1876–1894) other than that the secondary data were available for these years from the particular series of yearbooks (*Öfversigt af Finska Vetenskaps-Societetens förhandlingar*) and, as such, form a homogeneous ensemble.

Site names were adopted from the county level, with a few exceptions from their corresponding latitude and longitude. Moreover, the observation dates originally given in the calendric format (month and day) were transformed to the number of days relative to the June solstice (June 20th, 21st or 22nd, depending on the year of observation). This is a method that accounts for potential bias, especially in the case of a long series of data, that results from the mismatch between the length of the solar year and the slightly longer average year in the Gregorian calendar (see Sagarin and Micheli 2001; Sagarin 2001, 2009). In practice, the date is presented as a positive or negative integer or zero. That is, negative values refer to dates predating the June solstice; the positive ones refer to dates postdating the solstice. A zero value is obtained when the observation was made on the day of the June solstice.

Phenological stages originally written in German, Finnish and/or Swedish were translated into English and standardized by excluding synonyms. All scientific species names written in Latin, Swedish and/or Finnish were checked for consistency. The phenological stage of haymaking was not attributed to any scientific names and is referred to as “hay”. The scientific names of species were changed when the names used in the data did not match the modern nomenclature. This work followed the nomenclature given in Hämet-Ahti et al. (1998). In some cases, it was not possible to ascertain which species was recorded, as the names may have changed since the original publications. For example, the rejected name *Betula alba* does not differentiate between *B. pubescens* and *B. pendula*.

### Outlier detection

To evaluate citizen science data, statistical detection of potential outliers was calculated for each observation date value as the z-score statistic, adopted from the methodology of Li et al. (2020), with slight modifications suggested by Holopainen et al. (2023) to account for the characteristics of Finnish historical datasets. First, to account for the latitudinal (poleward decrease in temperature) trends in the dates of the same phenological event across the country, the dates of a phenological event (e.g., leaf outbreak of *Betula* sp.) were explained by the latitudes of their observation sites using linear regression. Previously, latitudinal relationships were observed by positive and negative slopes for the events occurring in spring and autumn, respectively (Holopainen et al. (2018). This accounted for the latitudinal (poleward decrease of temperature; Laaksonen 1976) trends in the dates of the same phenological event across the country. Second, the residuals from the latitude-dependent trendline were calculated as the difference between the observed dates and those expected by the linear model. Third, z-scores were produced by dividing each residual by the residuals’ standard deviation. Fourth, the mean time series of z-scores were calculated for events of which median dates had occurred before, during and after the June solstice. The corresponding yearly mean values were subtracted from the original z-scores to obtain a new set of z-scores which are reported in this study. We adopted the values of the slope and that of the residuals’ standard deviation, as well as the mean time series of z-scores, from the study of Holopainen et al. (2023) due to a higher number of observations from the same region and thus a more representative collection of phenological observations in that study. This estimation was repeated separately for the dates of each phenological event with at least 30 observations to calculate the slope (see above). According to Li et al. (2020), the dates with z-scores above 3.0 or below -3.0 could be suspected as potential outliers. In this dataset, we did not intend to remove any observations but to compare the distributions of z-scores of data obtained from formal notebooks and historical publications. In so doing, the number of observations with a z-score above 3.0 or below -3.0 was determined for both primary and secondary data. This comparison was made for phenological events with at least 30 observations.

### Secondary data

Data from the phenological diaries or notebooks were compared with similar data from historical yearbooks. Plant phenological data from publications presenting observations from 1876–1894 were analysed. These publications represent the compilations of data made public by Moberg (1878a, b, 1879, 1880, 1881, 1882, 1883, 1884, 1885b, 1886, 1887, 1888, 1889, 1890, 1891, 1892, 1893, 1894b) with data from 1876 to 1893 and by Kihlman (1895) with data from 1894. These published works provided the information used to create this dataset, with limited details (in Swedish and German) and guidelines outlining how the data was collected. Observations were made daily through the growing season and were likely dependent on the opportunities in the volunteers’ daily lives. These data were adopted from Holopainen et al. (2023). Similar to the primary data, the observation sites were obtained from the county level, with corresponding coordinates. Moreover, the dates were transformed to the number of days relative to the June solstice. Phenological stages were transformed into English, and outdated taxonomic names were updated as needed. Potential outliers were detected using the method described above regarding primary data.

### Comparison of the datasets

The primary and secondary data were visually and statistically compared. First, photographs illustrating the variety of phenological diary entries (primary data) were shown in combination with a journal pages with phenological data (secondary data). Second, the number of phenological observations was determined for primary and secondary data. Third, the sites with available primary and secondary data were plotted on a map. Fourth, the number of phenological observations in the primary and secondary data was determined for each month. Fifth, temporal variations in the number of sites, taxa, phenological stages/substages and events were determined to compare primary and secondary data. Sixth, the distribution of the dates (their z-scores, see above) of the primary and secondary data were compared for kurtosis and skewness (Sokal and Rohlf 1981) of and for the percent of observations with |z|> 3.

## Results

### Inspecting the notebooks

**Fig. 1:**
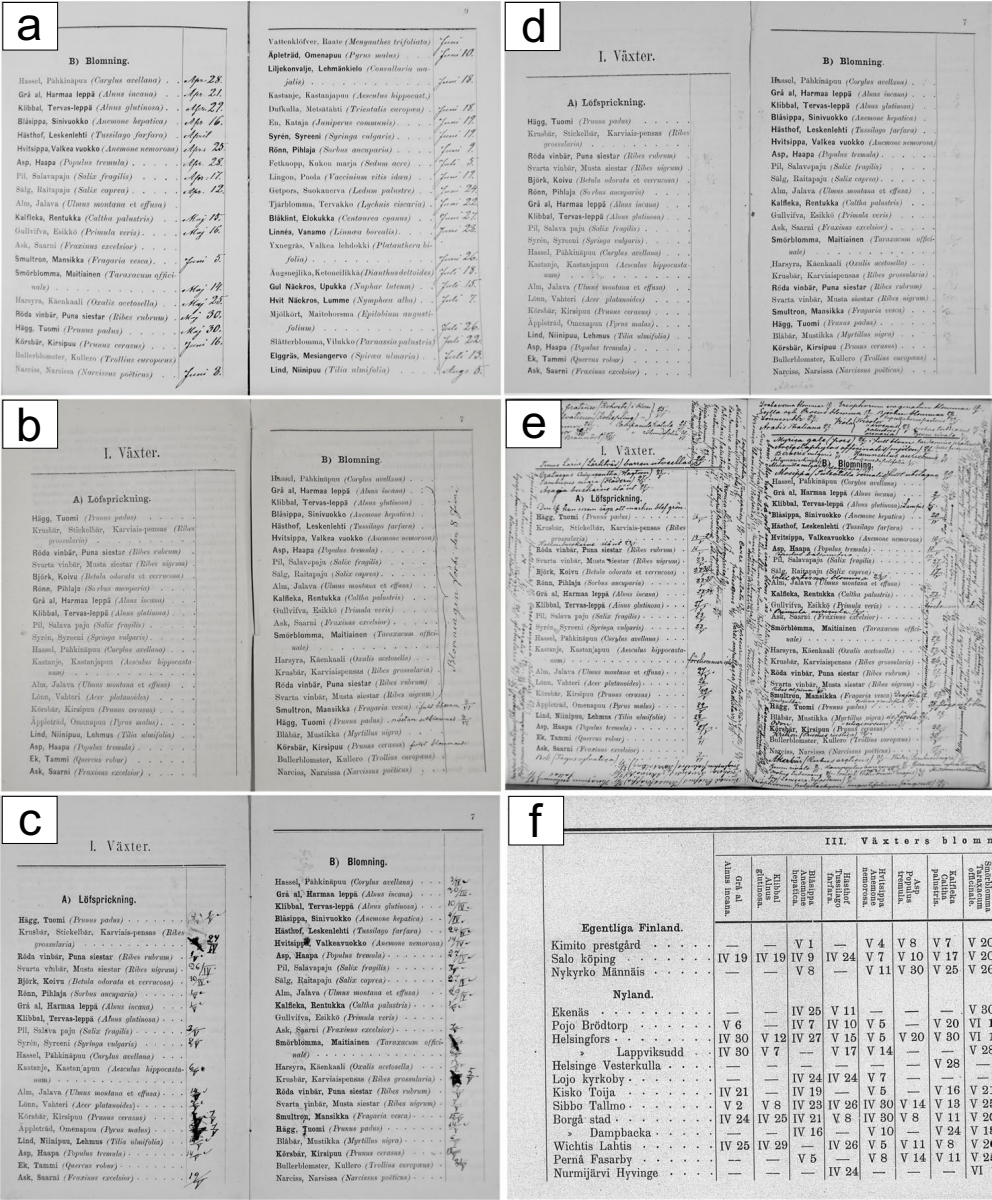
Documentary evidence available from formal phenological diaries or notebooks (primary data) and historical publications (secondary data) depicted using photographic images. Photographs of phenological notes with excellent handwriting (a), inaccurately/verbally described dates (b), ink that had smeared (c), pencil-written faint text (d) and that with a large colume of information (e), contrasted with a view from historical publication (f). All photos (a–f) by Jari Holopainen

The phenological diaries or notebooks from 1876 to 1894 are small multipage booklets with a printed list of plant names and phenological stages/substages and a blank space for dates to be written by the observer (Fig. 1a). The data obtained from these booklets were used as our primary data. The total number of booklets inspected in this study was 1846. Two booklets did not contain markings regarding the year when the observations were made. The data in these booklets were not included in the present study. After their removal, the number of booklets used was 1844. In rare cases, the event date was described verbally (Fig. 1b). Such descriptions could denote, for example, that the event had taken place “at the beginning of June” or “before the end of October”. Data from these observations were not included in the dataset. There were also cases where the handwriting was not clear, or the ink had smeared (Fig. 1c). The use of pencil, on the other hand, resulted in faint text in some cases (Fig. 1d). Observations with such readouts were not included in the dataset when the writing remained undiscernible. The exact number of observations rejected, for reasons discussed above, was not recorded but is estimated to be significantly less than one percent. All the other observations were included in the dataset. Commonly, the volunteers did not observe all requested events; however, some booklets contained information on events not requested in the booklet. Occasionally, the amount of extra information was substantial (Fig. 1e). These extra data were systematically included in the dataset similarly to the required data.

### Characterization of the primary data

**Fig. 2:**
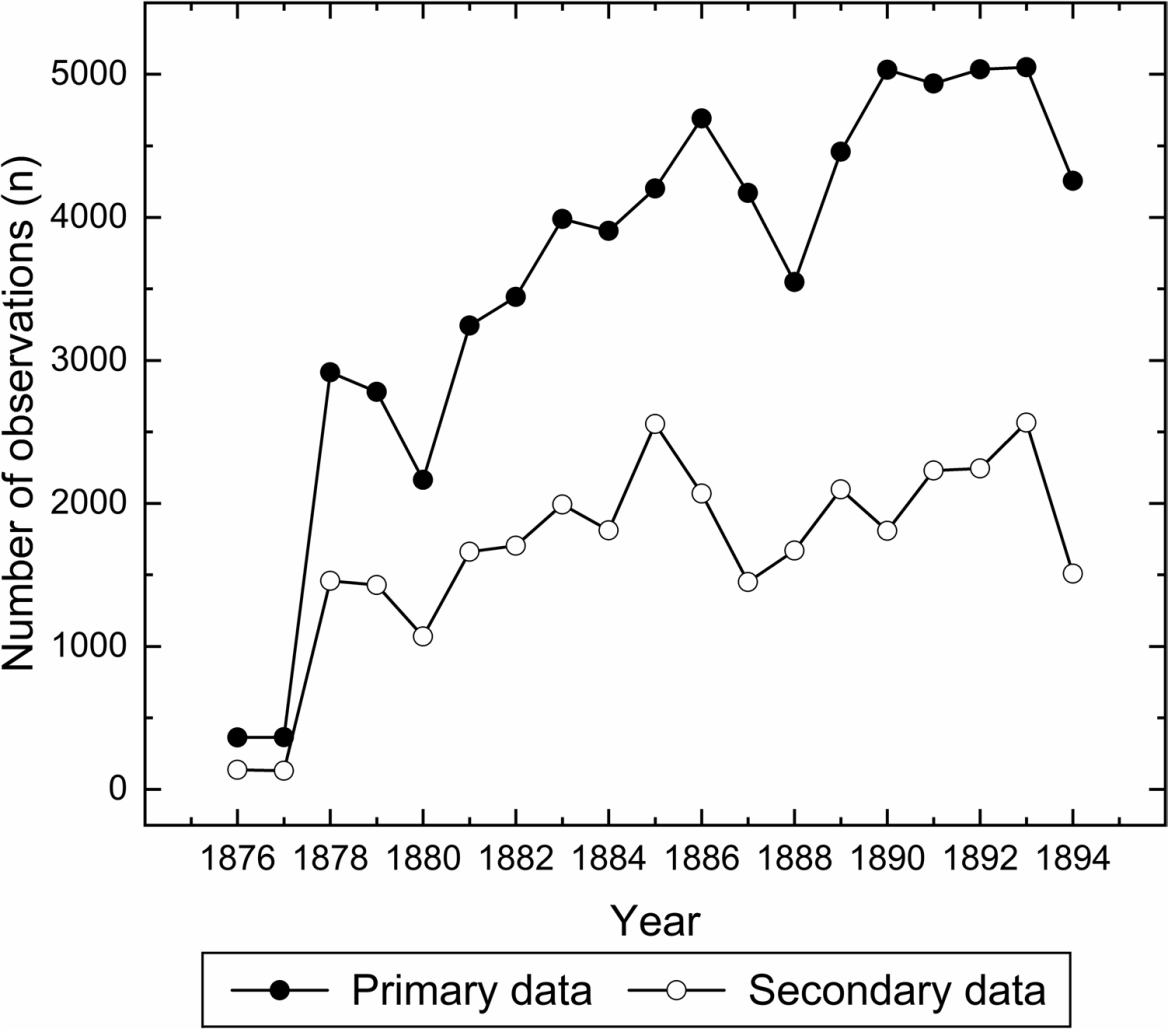
Temporal variations in the number of phenological observations in the primary and secondary data. See Table S1 for the data values

Four taxon observations could not be identified due to inconsistent abbreviations/incomplete notes. These observations were not included in the present dataset. After this removal, the data contained 68547 observations with an identified site and date, species, and phenological stage/substage. The scientific names of species were modified in the case of 25679 observations. The number of observations per year ranged between 364 and 5050 (Fig. 2). Generally, the number of observations per year increased over the study period.

**Fig. 3:**
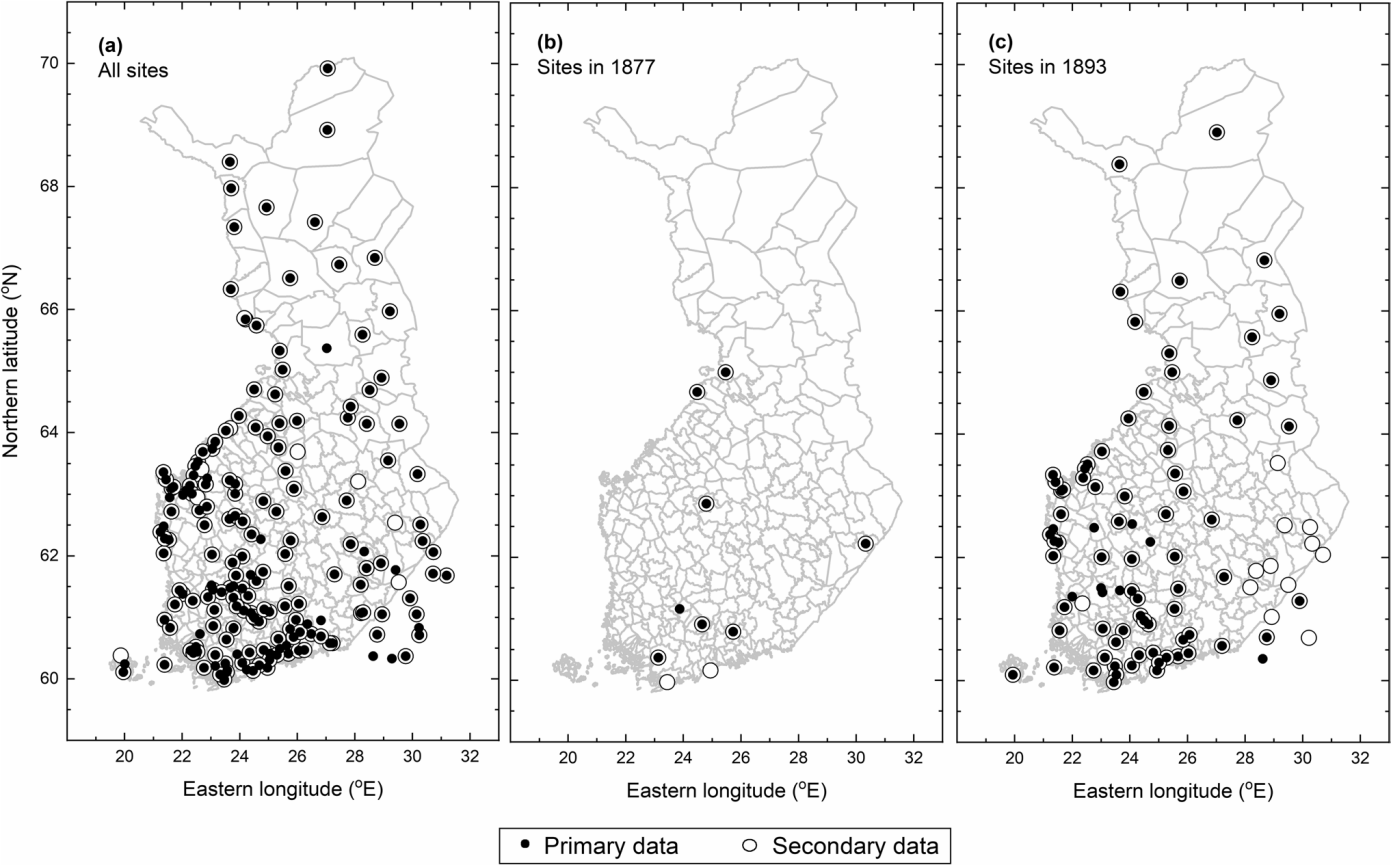
A map of Finland with phenological observation sites. The sites with data are shown over the entire period (1876–1894) (a), and during 1877 when the number of sites was low (b) and 1893 when the number of sites was high (c). Filled and open circles denote the sites with available data from the primary and secondary data, respectively

In total, there were 183 sites (see Fig. 3a). The number of sites increased from 10 to approximately 70–80 over the 19-year study period. During the early years (1876–1877), the sites were spread over the southern half of the country (Fig. 3b). Since 1878, when the collection of data started to increase, there were sites across the country; however, locations were concentrated in the southern and western portions of the country (Fig. 3c). This general trend is also reflected in the latitudes and longitudes of all the observations and averaged 61.84°N (SD = 1.62°) and 25.34°E (SD = 2.63°), respectively.

**Fig. 4:**
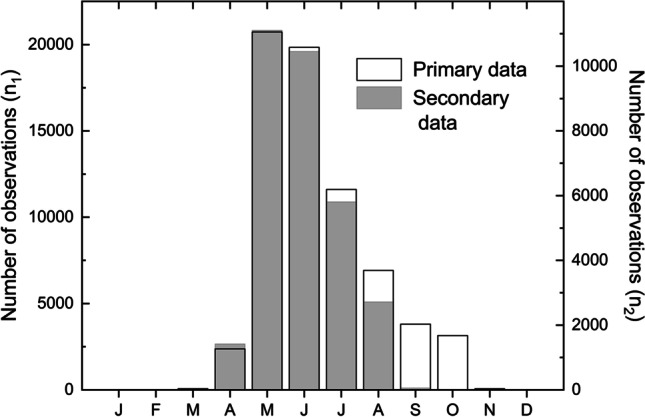
Monthly availability of phenological observations in the primary (n_1_) and secondary data (n_2_). See Table S2 for the data values

Most observations were made in May and June (Fig. 4). More than half of the data (59%) were recorded during these two months. Moreover, the remaining summer months, July and August, were also covered but to a lesser degree (10% and 17%, respectively). The months surrounding this late spring–summer season, April, September and October, each represented 3–6% of observations. There were less than 100 observations made in other months, except in January (with no observations).

**Fig. 5:**
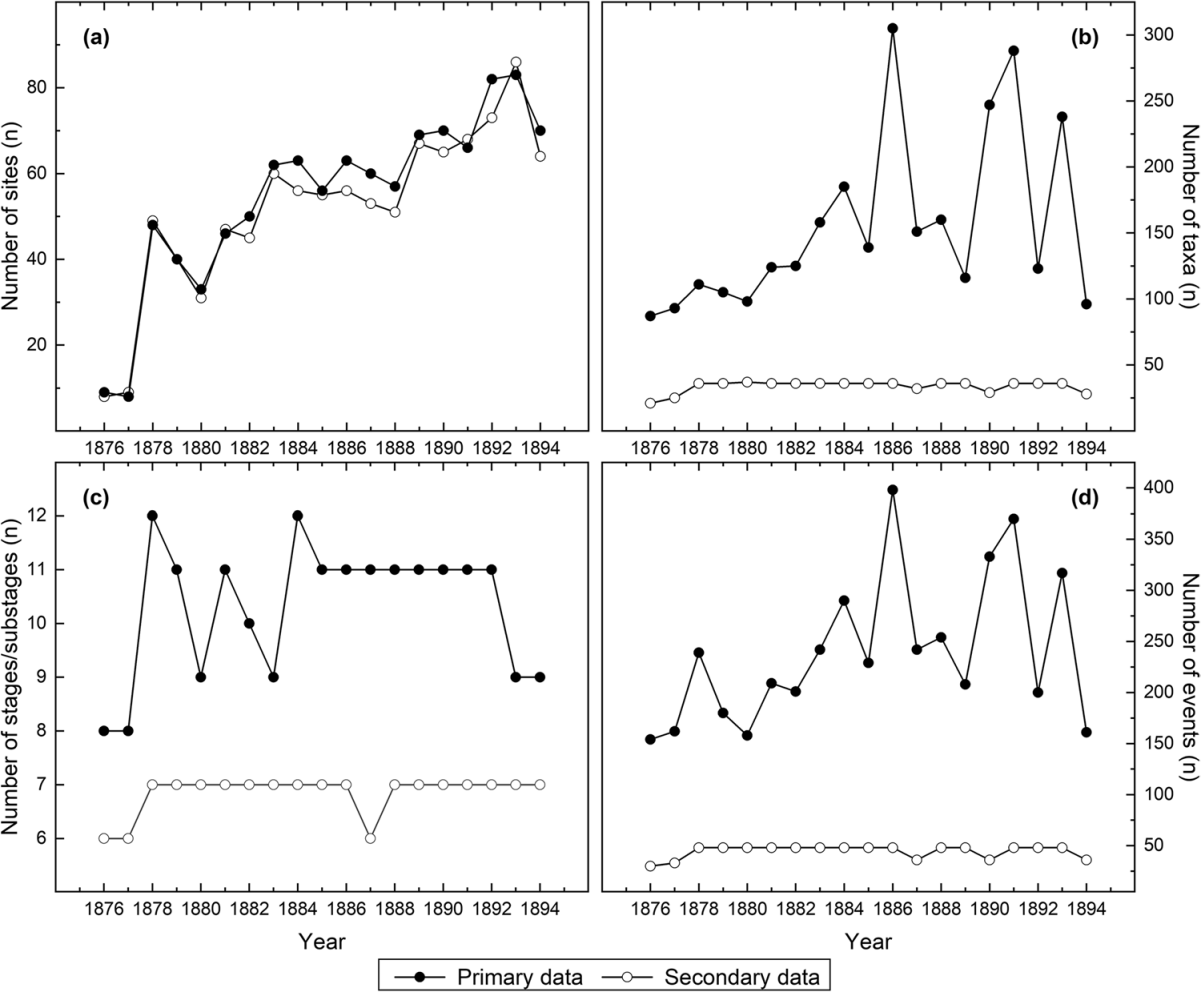
Temporal variations in phenological observations. Variations are shown for the primary and secondary data and given as the numbers of sites with available data (a), observed taxa (b), stages/substages (c), and phenological events (d). See Table S3 for data values

There were 513 taxa identified to the species or genus level. The five most common species were bird cherry (*Prunus padus*), rye (*Secale cereale*), rowan (*Sorbus aucuparia*), barley (*Hordeum vulgare*), and oat (*Avena sativa*). Generally, the species list mirrors the importance of agricultural activities during the observational timeframe. The number of sites (Fig. 5a) and observed taxa varied considerably through time (Fig. 5b). The number observed increased from approximately one hundred to two hundred until 1884, after which date the changes were larger from one year to the next, between 100 and 300.

The primary data contained observations on 12 different phenological stages/substages. The five most common stages/substages were flowering, leaf outbreak, fruit ripening, the start of sowing, and the mid-stage of leaf fall. The variety of the stages showed that common observations are distributed over the different seasons and contain information on both natural and human-related phenomena. The number of observed stages/substages exhibited changes from a minimum of 8 to a maximum of 12 (Fig. 5c).

As a result, the primary data contained information on 727 different phenological events. The five most common events were the flowering of *Prunus padus*, the leaf outbreak of *Betula* sp., the leaf outbreak of *Sorbus aucuparia*, the flowering of *Sorbus aucuparia*, and the leaf outbreak of *Prunus padus*. The number of events increased from around 150 to 300 until 1884, after which the changes were, similar to the number of observed taxa, more sizeable from year to year (Fig. 5d). It appeared that the number of events resulted predominantly from the number of observed taxa.

### Comparison between the primary and secondary data

The secondary data were available in the form of well-organised species lists, their phenological stages and dates (Fig. 1f). This data contained 31589 plant phenological observations. This number is less than half (46.1%) of the observations made in the primary data. In the secondary data, the number of observations per year ranged between 138 and 2564 (Fig. 2). The total number of sites was 165 (see Fig. 3). In the secondary data, the mean latitude and longitude of all the observations were 61.93°N (SD = 1.69°) and 25.39°E (SD = 2.60°), which were relatively similar to those of the primary data. Most (85%) of the sites were included both in the primary and secondary data. Interestingly, there were cases when observations from a particular site were included in the secondary data but not in the primary data (see Fig. 3).

The secondary data contained relatively more observations in May and June than the primary data (Fig. 4). Moreover, there was a considerable reduction of autumnal observations in the secondary data, where September and October observations accounted for less than 1% of the total observations. However, in the primary data, over a tenth of the observations (10.1%) were made during these two months.

There was a considerable reduction in the number of taxa included in the secondary data, totalling 42. The most common species were rye, Nordic currant (*Ribes spicatum*), rowan, bird cherry, and European strawberry (*Fragaria vesca*).

The five most common stages/substages in the secondary data were flowering, leaf outbreak, fruit ripening, sowing, and the start of ear formation. The secondary data contained observations on nine different phenological stages/substages. The five most common events were the start of ear formation of rye, the flowering of rye, the sowing of oat, the leaf outbreak of birch and the harvest of rye. The number of different phenological events in the secondary data was 68. The number of events was reduced, and the number of (selected) taxa, stages, and events was more consistent through the study period in the secondary data (Fig. 5b–d).

**Fig. 6:**
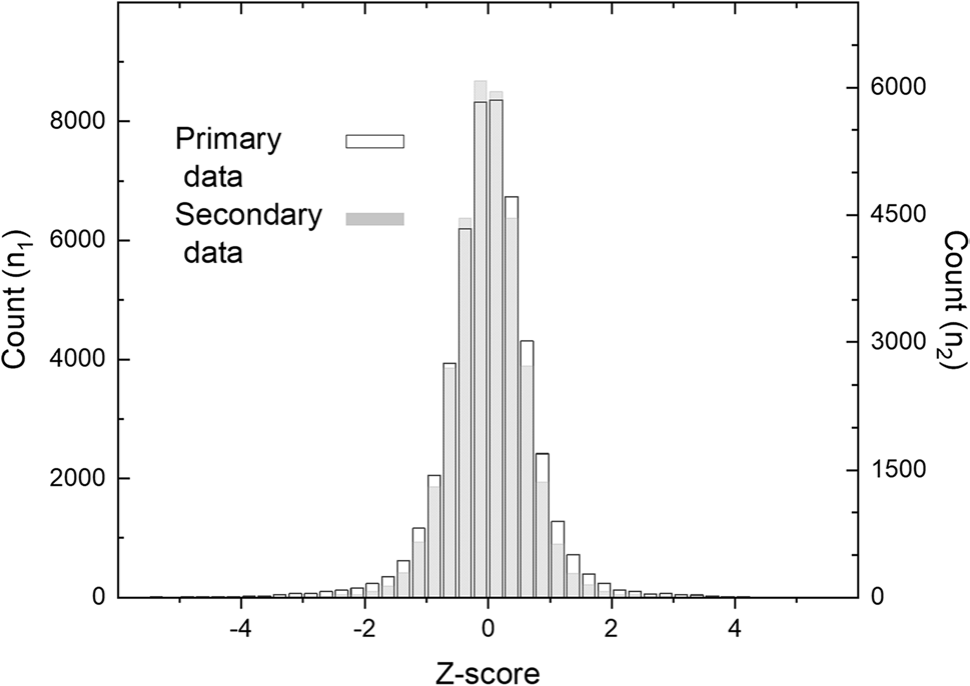
Distributions of dates from the primary (n_1_) and secondary data (n_2_) are given as z-scores. See Table S4 for data values

Comparison of the distributions of the dates showed a higher count of very positive or negative z-scores (here, |z|> 3) for the primary data relative to the secondary data (Fig. 6). This difference was reflected in the kurtosis of the two data which were 22.6 and 6.6 for the primary and secondary data, respectively. The skewness of the two data was relatively similar, with estimates of -0.19 and -0.12, respectively. Such leptokurtic distributions indicate that the observations are accumulated near the mean and at the distribution’s tails, with fewer observations in the intermediate regions (Sokal and Rohlf 1981). This phenomenon was stronger for the primary data. The percent of observations with |z|> 3 for the primary and secondary data were 1.5% and 0.37%, respectively. These results suggest that the secondary data contained a smaller percentage of potential outliers, according to the criteria set by Li et al. (2020).

## Discussion

It appears that the phenological observations available in the primary data (original phenological diaries) were heavily screened before publishing the secondary data (publications). Less than half the number of original observations made by the volunteers were eventually published by the Finnish Society of Sciences and Letters (Moberg 1878a, b, 1879, 1880, 1881, 1882, 1883, 1884, 1885b, 1886, 1887, 1888, 1889, 1890, 1891, 1892, 1893, 1894b; Kihlman 1895). This reduction was heavily seen in the number of observed taxa, which dropped from 511 (primary data) to 42 (secondary data). Such alterations likely demonstrate the attempts of the Society to standardize the datasets for their published, tabulated form. Practically, the standardization resulted in a relatively consistent number of taxa, as evident from the secondary data, with only slight variations from year to year (Fig. 5b). Similar standardization is evident regarding phenological stages, albeit to a lesser degree (Fig. 5c). This initial data standardization has direct implications for its reuse. Less available data is presented, and it is presented in a more accessible form, making the process of digitization less laborious and time-consuming. The accessibility is further increased by typesetting with no complications due to poor handwriting or problems with ink smearing.

A closer inspection of the data properties indicated that the reduction of stages in secondary data mainly concerned those observed during the latest part of the growing season, typically in September and October. Consequently, the proportion of data representing late-spring/early-summer phenology increased (Fig. 4). This suggests that choosing which data to publish was not only to standardize the published dataset but to increase information on plant growth early in the growing season. This emphasis likely reflects the practical reasons for collecting plant phenological data in Finland and, therefore, the needs the data hoped to fulfil. More specifically, it was hoped that a deeper understanding of nature and its cycles could help predict future climates and agricultural distress (Holopainen et al. 2023) and that spring is crucial for agricultural success. The “terrible famine year” in Finland following the crop failure of 1867, which increased the mortality rate to 79 per one thousand in 1868 (Jutikkala and Kauppinen 1971), was triggered by a cold spring season. The severity was so extreme that the estimated statistical probability for such a low May temperature was estimated to be about 1/500 (Jantunen and Ruosteenoja 2000). We infer that Adolf Moberg, the professor at the Imperial Alexander University of Finland, who had also compiled a large amount of eighteenth and nineteenth century phenological observations (Moberg 1857, 1860, 1885a, 1894a), must have had these linkages in mind when collating and editing the dataset for its published form. Similarly, this could be the reason for disregarding much of the autumn phenology, i.e., observations made after the grain harvest, when selecting which data was to be published. The emphasis on understanding agricultural processes was similarly evident in the selection of phenological events included in the secondary data (see above): four of the five most frequently published events were related to rye and oat, whereas the five most commonly observed events in the primary data pertained to non-agricultural, arboreal species (bird cherry, birch and rowan).

We note that the emphasis on agricultural events in the historical data contradicts recent suggestions to exclude plant phenological data that explicitly deal with agricultural information, such as when a crop was harvested (Stucky et al. 2018). While such data is also connected to human activities and are not purely naturally linked to the timing of plant life-cycle events, agricultural data should not, in our understanding, be disregarded. Our historical examples show the connections of agricultural phenology to food production in the past. Moreover, recent reports continue to use agricultural phenological data to evaluate disrupted crop growth and development and their economic yield due to the ongoing climatic changes, to which farmers must adapt (Tao et al. 2006; Chevet et al. 2011; Fatima et al. 2020). Similar to the viewpoints maintained by early scholars in Finland, this literature shows the sustained use of agricultural phenology data collection and the importance such data can have in estimating the climate-related risks to farming. They also demonstrate the potential relationships between climatic variations and markets of various agricultural products. Contrastingly, the varying viewpoints demonstrate the subjectivity of both historical and current researchers, which may similarly continue to influence how the databases are built and how the data are used in future applications.

Silvertown (2009) states that a citizen scientist is a volunteer who collects data, potentially with professional counterparts, on a scientific project. He also highlighted that the roots of citizen science go back to the very beginnings of modern science itself. This is also the case of the Finnish phenological dataset, which began in the middle of the eighteenth century (Elfving 1938; Terhivuo et al. 2009; Holopainen et al. 2012), following Linné’s recommendations to develop phenological networks (Dahl and Langvall 2008). Since then, the task has been pursued by volunteers and early naturalists who, in the past, represented the educated class of society (Elfving 1938). According to Holopainen et al. (2018), their titles typically included occupations such as vicars, priests, professors, doctors, rural police chiefs, lieutenants, doctors and students. On these grounds, the observations made could be seen as a representation of citizen science data. The opportunistic variations reflected in the initial observations, or primary data, exhibit the nature of citizen science. These variations are demonstrated by notable temporal changes in the number of observed species and their phenological stages (Fig. 5). Generally, the existence of this dataset illustrates the importance of citizen science in gathering large amounts of data in which its scientific value continues to increase as long as its collection continues. More specifically, the figures also demonstrate the hardships supporting scientific targets that are fed by the enthusiasm or excitement of citizen scientists.

The number of sites increased abruptly from 1877 to 1878 (Fig. 5a). This increase was likely a result of changes made in the formal notebooks sent by the Society to volunteers in 1878. The increase occurred when the notebook format was altered by decreasing the number of inquired species and their phenological stages. Moreover, the booklet contained no more entry spots for meteorological observations, some of which were previously demanded from the volunteers. In practice, the change of the booklet reduced the number of pages to 16 (it is noteworthy that the booklets also contained entries for animal phenology, not included in the present study). This was a sizeable change as the booklet of the preceding years had considerably more pages. The Swedish version from 1846 contained 70 pages and 60 pages in 1856, and the (first) Finnish version had 58 pages in 1861. The less demanding task was evidently more attractive to the volunteers, with an apparent renewal of excitement in making observations and returning the notebooks to the Society (Elfving 1938; Holopainen et al. 2012). Even so, the percentage of returned notebooks remained low: it is known that as many as 1000 copies of the booklet were printed in 1878 (Elfving 1938). In any case, the high number of returned notebooks also increased the number of sites in the secondary data. The number of sites in the primary and secondary data are similar, which likely demonstrates the emphasis of the Society to spatially cover the country as densely as possible. A closer look at the sites shows that not all sites present in the secondary data were present in the primary data (Figs. 3 and 5a). The number of missing sites (equalling the number of returned notebooks) was low but supports the idea that not all notebooks returned to the Society were available at the National Archives of Finland. We have no further information to explain the discrepancy, but we speculate that some booklets may have been lost before archiving.

The ‘start of sowing’ and ‘sowing’ were among the most common stages/substages in the primary and secondary data, respectively. Disregarding the taxa, the ‘start of sowing’ was recorded 5183 times in the primary data, whereas ‘sowing’ was recorded only 93 times. However, in the secondary data, ‘sowing’ was recorded 2615 times, with no recorded stages denoted as the ‘start of sowing’. Calculating the mean dates for oat and barley could further explain this issue. In the primary data, the mean dates for the ‘start of sowing’ of oat and barley were -42.4 (days before the June solstice) and -28.5, respectively. In the secondary data, contrastingly, the respective mean dates for the ‘sowing’ were -42.7 and -28.2. Combined, it is evident that the sowing dates have been published without mentioning the substage, start (“börjar” in Swedish, “alku” in Finnish), to which it was commonly referred by the observers. These examples demonstrate the discrepancies research may face when dealing with information that is not directly available from the original sources, as the editing of the dataset may have masked some of its original details. This could result in hidden layers of information which are not directly evident from the (secondary) sources used for digitizing the data. More precisely, the comparison indicates the presence of uncertainties related to substage identification in the data from the publications of the Finnish Society of Sciences and Letters. Moreover, the outcomes demonstrate a need for more detailed analyses of seasonal plant development, regarding substages, especially in association with the phenology of agriculture.

Our examples of data editing originate from historical scientific publications. Similar sources of historical phenological data, i.e., yearbooks, monthly climate reviews and/or weekly bulletins of the national or local institutes, have been previously highlighted by Koch et al. (2007) and more recently detailed by Ovaskainen et al. (2020) and Kalvāne et al. (2021). Apart from scientific reports, phenological observations can be collected from newspaper articles where seasonal plant development and events may have been highlighted frequently in the past (Aono and Saito 2010; Moreno et al. 2016; Fitchett and Raik 2021). Although an approach to scan old newspapers may not result in abundant data, the peculiarities of edited information will likely remain. Our comparison using primary and secondary data showed that the screening of primary data can be sizeable even in scientific publications. Similar to our setting, historical journalists have likely been supported by citizens reporting phenological events to editing offices. However, the offices may have edited the information and published what they have deemed most interesting for the readership; for example, it could be hypothesised that the abnormal events remain overrepresented. Selective reporting of recent climatic and environmental events has been shown to exist in modern newspapers. Typically, the media focuses on negative effects (King et al. 2019; Feldman and Hart 2021). Our study illustrates the relevance of considering the aspects of screening when secondary sources of useable scientific data are being collected and used in scientific analyses. Such approaches can prevent us from obtaining the full spectrum of data available in the primary sources.

The quality of the citizen science observations, and their reliability for scientific analyses, have been hypothetically questioned in several plant phenological studies (Fuccillo et al. 2015; Bison et al. 2019; Li et al. 2020). In this study, the changes in the statistical distribution between primary and secondary data were shown (Fig. 6). These changes demonstrate that the original phenological observations were screened for outliers by the compilers of the Society’s publications. This alteration has reduced the number of observations with excessively positive or negative z-score values; this change is further reflected in the kurtosis of the two data. In this study, the value of |z|> 3 was used as a threshold for potential outliers, as suggested by Li et al. (2020), with slight modifications to obtain z-scores for the present sample of observations (Holopainen et al. 2023). We note that there are other applications for outlier evaluation in phenological datasets (Linkosalo et al. 1996). The method we used was chosen based on the comparison of different outlier detection techniques, and this method was evaluated to be a simple and effective method for improving the reliability of citizen science phenology observations (Li et al. 2020). We are not aware of any statistical methods used by compilers of the secondary to pinpoint outliers (Moberg 1878a, b, 1879, 1880, 1881, 1882, 1883, 1884, 1885b, 1886, 1887, 1888, 1889, 1890, 1891, 1892, 1893, 1894b; Kihlman 1895). Possibly, the dates were screened qualitatively by scanning with the eye and looking for suspiciously early or late dates. The statistical distributions indicate that the secondary data compliers of the secondary data were eventually aware that the original data might contain values not representative of the sample. It revealed that they successfully removed observations potentially representing outliers in terms of the modern statistical criteria used in this study. This historical process aimed to publish a coherent phenological dataset indicative of systematic responses to changing seasons, with high relevance to agricultural and natural sciences. Intriguingly, the secondary data compliers approach concurs with the view provided by modern statistics. This demonstrates that removing outliers can effectively improve the reliability of phenological data (Linkosalo et al. 1996; Li et al. 2020). It is possible that the screening was carried out at least until 1893 by Professor Adolf Moberg himself, for whom Finnish phenology is known to have represented a lifetime vocation (Elfving 1938).

## Conclusions

There are many choices for producing and publishing phenological data. Relying on citizen science, observations made by volunteers represent primary data that may reflect opportunistic variations. Our findings from historical Finland demonstrated notable yearly changes in the observations. Publishers of the observations took these peculiarities into account when reproducing the data for future generations of researchers. In our case, the primary data were heavily screened before publication, resulting in the secondary data. The findings of our study could be summarized as follows:

A) Less than half of the primary data were eventually published (as secondary data); this reduction primarily concerned the number of observed taxa and, to a lesser degree, the number of phenological stages. The primary data had also been screened for potential outliers. The screening did not largely affect the number of sites.
B) These alterations demonstrate an attempt to standardize the data. However, the choices made by the historical actors were not neutral and reflected their own criteria and preference. In the case of historical Finland, the aim was to collect data to support agricultural decision-making and create a focus on spring events at the cost of autumn phenology.
C) While the well-organised typesetting of the secondary data makes digitization less laborious to re-users, the current researchers ought to be aware of how the editors of the secondary data may have carried out their own reshaping. A fuller spectrum of observations is available in the primary data. This shows the importance of storing the original diaries.

It is evident that current researchers make their own choices when selecting which part of the data will be included in their inquiries. Moreover, the media coverage of current environmental phenomena focuses largely on extreme and hazardous events. These choices continue to affect the ways we (re)generate phenological information.

## Supplementary Information

Below is the link to the electronic supplementary material.

Supplementary file1 (PDF 442 kb)

## References

[CR1] Aono Y, Saito S (2010). Clarifying springtime temperature reconstructions of the medieval period by gap-filling the cherry blossom phenological data series at Kyoto, Japan. Int J Biometeorol.

[CR2] Bison M, Yoccoz NG, Carlson BZ, Delestrade A (2019). Comparison of budburst phenology trends and precision among participants in a citizen science program. Int J Biometeorol.

[CR3] Brereton TM, Botham MS, Middlebrook I, Randle Z, Noble DG, Roy DB (2014) United Kingdom butterfly monitoring scheme report for 2013. Centre for Ecology and Hydrology and Butterfly Conservation

[CR4] Burris G, Washburn J, Lasheen O, Dorribo S, Elsner JB, Doel RE (2019). Extracting weather information from a plantation document. Clim Past.

[CR5] Chevet J-M, Lecocq S, Visser M (2011). Climate, grapevine phenology, wine production, and prices: Pauillac (1800–2009). Am Econ Rev.

[CR6] Cook BI, Wolkovich EM (2016). Climate change decouples drought from early wine grape harvests in France. Nat Clim Chang.

[CR7] Courter JR, Johnson RJ, Stuyck CM, Lang BA, Kaiser EW (2013). Weekend bias in Citizen Science data reporting: implications for phenology studies. Int J Biometeorol.

[CR8] Dahl Å, Langvall O (2008) Observations on phenology in Sweden — past and present. In: Nekovář J (ed) The History and Current Status of Plant Phenology in Europe. COST Action 725, Vammala, pp. 161–165

[CR9] Elfving F (1938) Fenologiset havainnot. Societas Scientiarum Fennica, Commentationes Humanarum Litterarum 10:204–213

[CR10] Elfving G, Mickwitz G (1988) Suomen Tiedeseuran kolmas puolivuosisata 1938–1987. Bidrag till kännedom af Finlands natur och folk 136b:1–207

[CR11] Fatima Z, Ahmed M, Hussain M, Abbas G, Ul-Allah S, Ahmas S, Ahmed N, Ali MA, Sarwar G, ul Haque E, Iqbal P, Hussain S (2020) The fingerprints of climate warming on cereal crops phenology and adaptation options. Sci Rep 10. 10.1038/s41598-020-74740-3 10.1038/s41598-020-74740-3 PMC7581754 33093541

[CR12] Feldman L, Hart PS (2021) Upping the ante? The effects of “emergency” and “crisis” framing in climate change news. Clim Chang 169. 10.1007/s10584-021-03219-5

[CR13] Fitchett JM, Raik K (2021) Phenological advance of blossoming over the past century in one of the world’s largest urban forests, Gauteng City-Region, South Africa. Urban For Urban Green 63. 10.1016/j.ufug.2021.127238

[CR14] Fuccillo KK, Crimmins TM, de Rivera CE, Elder TS (2015). Assessing accuracy in citizen science-based plant phenology monitoring. Int J Biometeorol.

[CR15] Häkkinen R (1999) Analysis of bud-development theories based on long-term phenological and air temperature series: application to Betula sp. leaves. Ph.D. Dissertation, Finnish Forest Research Institute, Research Papers 754, 59 p

[CR16] Häkkinen R, Linkosalo T, Hari P (1995). Methods for combining phenological time series: application to bud burst in birch (*Betula pendula*) in Central Finland for the period 1896–1955. Tree Physiol.

[CR17] Häkkinen R, Linkosalo T, Hari P (1998). Effects of dormancy and environmental factors on timing of bud burst in *Betula pendula*. Tree Physiol.

[CR18] Hämet-Ahti L, Suominen J, Ulvinen T, Uotila P (1998) Retkeilykasvio (Field Flora of Finland), 4th edn. Finnish Museum of Natural History, Botanical Museum, Helsinki, 656 p

[CR19] Hari P, Aakala T, Hilasvuori E, Häkkinen R, Korhola A, Korpela M, Linkosalo T, Mäkinen H, Nikinmaa E, Nöjd P, Seppä H, Sulkava M, Terhivuo J, Tuomenvirta H, Weckström J, Hollmen J (2017) Reliability of temperature signal in various climate indicators from northern Europe. PLoS One 12. 10.1371/journal.pone.0180042 10.1371/journal.pone.0180042 PMC5491121 28662166

[CR20] Heikinheimo M, Lappalainen H (1997). Dependence of the flower bud burst of some plant taxa in Finland on effective temperature sum: implications for climate warming. Ann Bot Fenn.

[CR21] Helama S, Tolvanen A, Karhu J, Poikolainen J, Kubin E (2020). Finnish National Phenological Network 1997–2017: from observations to trend detection. Int J Biometeorol.

[CR22] Holopainen J (2004) The early climatological records of Turku. Finnish Meteorological Institute Reports 8, Edita, Helsinki, 59 p

[CR23] Holopainen J, Helama S, Timonen M (2006). Plant phenological data and tree-rings as palaeoclimate indicators since AD 1750 in SW Finland. Int J Biometeorol.

[CR24] Holopainen J, Gregow H, Helama S, Kubin E, Lummaa V, Terhivuo J (2012). History of Finnish plant phenological observations since the 1750s. Sorbifolia.

[CR25] Holopainen J, Helama S, Lappalainen H, Gregow H (2013). Plant phenological records in northern Finland since the 18^th^ century as retrieved from databases, archives and diaries for biometeorological research. Int J Biometeorol.

[CR26] Holopainen J, Helama S, Väre H (2018). Digitizing the plant phenological dataset (1750–1875) from collections of Professor Adolf Moberg: towards the development of historical climate records. Agric For Meteorol.

[CR27] Holopainen J, Helama S, Väre H (2023). Plant phenological dataset collated by the Finnish Society of Sciences and Letters. Ecology.

[CR28] Hox J, Boeije H (2005). Data collection, primary versus secondary. Encycl Soc Meas.

[CR29] Leche I, Hjelt O (1889) Förteckning på tiden, då de Allmænnaste træ och buskar kring Åbo utslagit blad och blommor åhren 1750, 51 och 52, enligen Kongl. Vet. Acad. begæran utrönt. Bidrag till kännedom af Finlands Natur och Folk 48:471–482

[CR30] Jantunen J, Ruosteenoja K (2000). Weather conditions in northern Europe in the exceptionally cold spring season of the Famine Year 1867. Geophysica.

[CR31] Jutikkala E, Kauppinen M (1971). The structure of mortality during catastrophic years in a pre-industrial society. Pop Stud.

[CR32] Kalvāne G, Kalvāns A (2021). Phenological trends of multi-taxonomic groups in Latvia, 1970–2018. Int J Biometeorol.

[CR33] Kalvāne G, Kalvāns A, Ģērmanis A (2021). Long-term phenological data set of multi-taxonomic groups, agrarian activities, and abiotic parameters from Latvia, northern Europe. Earth Syst Sci Data.

[CR34] Kihlman AO (1895) Sammandrag af de klimatologiska anteckningarna i Finland år 1894. Öfversigt af Finska Vetenskaps-Societetens förhandlingar 37:245–270

[CR35] King N, Bishop-Williams KE, Beauchamp S, Ford JD, Berrang-Ford L, Cunsolo A, Harper SL, IHACC Research Team (2019). How do Canadian media report climate change impacts on health? A newspaper review. Clim Chang.

[CR36] Kobori H, Dickinson JL, Washitani I, Sakurai R, Amano T, Komatsu N, Kitamura W, Takagawa S, Koyama K, Ogawara T, Miller-Rushing AJ (2016). Citizen science: a new approach to advance ecology, education, and conservation. Ecol Res.

[CR37] Koch E, Bruns E, Chmielewski FM, Defila C, Lipa W, Menzel A (2007) Guidelines for plant phenological observations. WMO/TD No. 1484. World Meteorological Organization, Geneva, 13

[CR38] Laaksonen K (1976). The dependence of mean air temperatures upon latitude and altitude in Fennoscandia (1921–1950). Ann Acad Sci Fennicae Series A III Geol Geogr.

[CR39] Lappalainen H, Heikinheimo M (1992) Relations between climatological and plant phenological observations. Survey of Plant Phenological Observations in Finland from 1896 to 1965, vol. 1. Meteorological Publications 20, 1–74

[CR40] Li JS, Hamann A, Beaubien E (2020). Outlier detection methods to improve the quality of citizen science data. Int J Biometeorol.

[CR41] Linkosalo T (2000). Mutual regularity of spring phenology of some boreal tree species: predicting with other species and phenological models. Can J For Res.

[CR42] Linkosalo T, Carter TR, Häkkinen R, Hari P (2000). Predicting spring phenology and frost damage risk of Betula spp. under climatic warming: a comparison of two models. Tree Physiol.

[CR43] Linkosalo T, Häkkinen R, Hari P (1996). Improving the reliability of a combined phenological time series by analyzing observation quality. Tree Physiol.

[CR44] Linkosalo T, Häkkinen R, Terhivuo J, Tuomenvirta H, Hari P (2009). The time series of flowering and leaf bud burst of boreal trees (1846–2005) support the direct temperature observations of climatic warming. Agric For Meteorol.

[CR45] Linkosalo T, Lappalainen HK, Hari P (2008). A comparison of phenological models of leaf bud burst and flowering of boreal trees using independent observations. Tree Physiol.

[CR46] Mayer A (2010). Phenology and citizen science: volunteers have documented seasonal events for more than a century, and scientific studies are benefiting from the data. Bioscience.

[CR47] Menzel A (2002). Phenology: its importance to the global change community. Clim Chang.

[CR48] Menzel A, Sparks T, Estrella N, Koch E, Aasa A, Ahas R, Alm-Kübler K, Bissolli P, Braslavska O, Briede A, Chmielewski FM, Crepinsek Z, Curnel Y, Dahl A, Defila C, Donnelly A, Filella Y, Jatczak K, Mage F, Mestre A, Nordli Ø, Penuelas J, Pirinen P, Remisova V, Scheifinger H, Striz M, Susnik A, van Vliet AJH, Wielgolaski FE, Zach S, Zust A (2006). European phenological response to climate change matches the warming pattern. Glob Chang Biol.

[CR49] Menzel A, Yuan Y, Matiu M, Sparks T, Scheifinger H, Gehrig R, Estrella N (2020). Climate change fingerprints in recent European plant phenology. Glob Chang Biol.

[CR50] Moberg A (1857) Naturhistoriska dag-anteckningar gjorda i Finland, Åren 1750–1845. Notiser ur Sällskapets pro Fauna & Flora fennica förhandlingar. Bihang till Acta Societatis Scientarium Fennicæ 3:95–250

[CR51] Moberg A (1860) Klimatologiska iakttagelser i Finland, föranstaltade och utgifna af Finska Vetenskaps-Societen. Första delen. År 1846–1855. I. Bidrag till Finlands naturkännedom, etnografi och statistic 7:1–361

[CR52] Moberg A (1878a) Sammandrag af de klimatologiska anteckningarne i Finland år 1876. Öfversigt af Finska Vetenskaps-Societetens förhandlingar 19:79–82

[CR53] Moberg A (1878b) Sammandrag af de klimatologiska anteckningarne i Finland år 1877. Öfversigt af Finska Vetenskaps-Societetens förhandlingar 20:118–121

[CR54] Moberg A (1879) Sammandrag af de klimatologiska anteckningarne i Finland år 1878. Öfversigt af Finska Vetenskaps-Societetens förhandlingar 21:261–279

[CR55] Moberg A (1880) Sammandrag af de klimatologiska anteckningarne i Finland år 1879. Öfversigt af Finska Vetenskaps-Societetens förhandlingar 22:155–167

[CR56] Moberg A (1881) Sammandrag af de klimatologiska anteckningarne i Finland år 1880. Öfversigt af Finska Vetenskaps-Societetens förhandlingar 23:101–113

[CR57] Moberg A (1882) Sammandrag af de klimatologiska anteckningarne i Finland år 1881. Öfversigt af Finska Vetenskaps-Societetens förhandlingar 24:89–107

[CR58] Moberg A (1883) Sammandrag af de klimatologiska anteckningarne i Finland år 1882. Öfversigt af Finska Vetenskaps-Societetens förhandlingar 25:158–176

[CR59] Moberg A (1884) Sammandrag af de klimatologiska anteckningarne i Finland år 1883. Öfversigt af Finska Vetenskaps-Societetens förhandlingar 26:193–216

[CR60] Moberg A (1885a) Klimatologiska iakttagelser i Finland, föranstaltade och utgifna af Finska Vetenskaps-Societen. Andra delen. År 1856–1875. I. Fenologiska anteckningar. Bidrag till kännedom af Finlands natur och folk 41:1–321

[CR61] Moberg A (1885b) Sammandrag af de klimatologiska anteckningarne i Finland år 1884. Öfversigt af Finska Vetenskaps-Societetens förhandlingar 27:111–129

[CR62] Moberg A (1886) Sammandrag af de klimatologiska anteckningarne i Finland år 1885. Öfversigt af Finska Vetenskaps-Societetens förhandlingar 28:115–133

[CR63] Moberg A (1887) Sammandrag af de klimatologiska anteckningarne i Finland år 1886. Öfversigt af Finska Vetenskaps-Societetens förhandlingar 29:217–242

[CR64] Moberg A (1888) Sammandrag af de klimatologiska anteckningarne i Finland år 1887. Öfversigt af Finska Vetenskaps-Societetens förhandlingar 30:111–135

[CR65] Moberg A (1889) Sammandrag af de klimatologiska anteckningarne i Finland år 1888. Öfversigt af Finska Vetenskaps-Societetens förhandlingar 31:183–206

[CR66] Moberg A (1890) Sammandrag af de klimatologiska anteckningarne i Finland år 1889. Öfversigt af Finska Vetenskaps-Societetens förhandlingar 32:151–175

[CR67] Moberg A (1891) Sammandrag af de klimatologiska anteckningarne i Finland år 1890. Öfversigt af Finska Vetenskaps-Societetens förhandlingar 33:235–259

[CR68] Moberg A (1892) Sammandrag af de klimatologiska anteckningarne i Finland år 1891. Öfversigt af Finska Vetenskaps-Societetens förhandlingar 34:309–333

[CR69] Moberg A (1893) Sammandrag af de klimatologiska anteckningarne i Finland år 1892. Öfversigt af Finska Vetenskaps-Societetens förhandlingar 35:155–180

[CR70] Moberg A (1894a) Fenologiska iakttagelser i Finland åren 1750–1845. Utgifna af Finska Vetenskaps-Societeten. Finska Litteratur-Sällskapets tryckeri, Helsingfors. Bidrag till kännedom af Finlands Natur och Folk 55:1–165

[CR71] Moberg A (1894b) Sammandrag af de klimatologiska anteckningarne i Finland år 1893. Öfversigt af Finska Vetenskaps-Societetens förhandlingar 36:199–229

[CR72] Moreno J, Fatela F, Moreno F, Leorri E, Taborda E, Trigo R (2016). Grape harvest dates as indicator of spring-summer mean maxima temperature variations in the Minho region (NW of Portugal) since the 19^th^ century. Glob Planet Chang.

[CR73] Ovaskainen O, Meyke E, Lo C et al (2020) Chronicles of nature calendar, a long-term and large-scale multitaxon database on phenology. Sci Data 7. 10.1038/s41597-020-0376-z 10.1038/s41597-020-0376-z PMC7012846 32047153

[CR74] Petrauski L, Owen S, Constantz G, Anderson J (2020). Developing a historical phenology dataset through community involvement for climate change research. Am J Clim Chang.

[CR75] Posthumus E, Crimmins T (2011). Nature's Notebook: a tool for education and research. Bull Ecol Soc Am.

[CR76] Sagarin R (2001). False estimates of the advance of spring. Nature.

[CR77] Sagarin R (2009). Using nature’s clock to measure phenology. Front Ecol Environ.

[CR78] Sagarin R, Micheli F (2001). Climate change in nontraditional data sets. Science.

[CR79] Sheail J (1980) Historical Ecology: The Documentary Evidence. Natural Environmental Council. Institute of Terrestrial Ecology. Cambridge, 21 p

[CR80] Silvertown J (2009) A new dawn for citizen science. Trends Ecol Evol 24:467–471 10.1016/j.tree.2009.03.017 19586682

[CR81] Sokal RR, Rohlf FJ (1981) Biometry. The Principles and Practice of Statistics in Biological Research, 2nd edn. W. H. Freeman and Company, San Francisco. 859 p

[CR82] Stucky BJ, Guralnick R, Deck J, Denny EG, Bolmgren K, Walls R (2018) The plant phenology ontology: a new informatics resource for large-scale integration of plant phenology data. Front Plant Sci 9. 10.3389/fpls.2018.00517 10.3389/fpls.2018.00517 PMC5938398 29765382

[CR83] Tao F, Yokozawa M, Xu Y, Hayashi Y, Zhang Z (2006). Climate changes and trends in phenology and yields of field crops in China, 1981–2000. Agric For Meteorol.

[CR84] Terhivuo J, Kubin E, Karhu J (2009). Phenological observations since the days of Linné in Finland. Ital J Agrometeorol.

[CR85] Vantieghem P, Maes D, Kaiser A, Merckx T (2017). Quality of citizen science data and its consequences for the conservation of skipper butterflies (Hesperiidae) in Flanders (northern Belgium). J Insect Conserv.

