# Supplementary material for: The written history of plant phenology: shaping primary sources for secondary publications
Source: Naturwissenschaften. 2023 Jul 6;110(4):34. doi: 10.1007/s00114-023-01861-w (PMC10326116; doi:10.1007/s00114-023-01861-w)
Supplement: Supplementary file 1 — Supplementary file1 (PDF 442 kb) [file 114_2023_1861_MOESM1_ESM.pdf]

Supplementary Information

The written history of plant phenology: shaping primary sources for secondary publications

Jari Holopainen, Samuli Helama and Henry Väre

- Table S1. Temporal variations in the number of phenological observations in the primary (n_1) and secondary data (n_2): data used in Figure 2
- Table S2. Monthly availability of phenological observations in the primary (n_1) and secondary data (n_2): data used in Figure 4
- Table S3. Temporal variations in phenological observations: data used in Figure 5
- Table S4. Distributions of the dates from the primary (n_1) and secondary data (n_2) given as z-scores: data used in Figure 6

Table S1. Temporal variations in the number of phenological observations in the primary (n_1) and secondary data (n_2). See Fig. 2 for plotted data.

Year	n_1	n_2
1876	364	138
1877	365	130
1878	2916	1458
1879	2779	1429
1880	2165	1069
1881	3244	1661
1882	3444	1704
1883	3989	1992
1884	3905	1811
1885	4202	2555
1886	4691	2068
1887	4171	1450
1888	3548	1670
1889	4458	2098
1890	5032	1808
1891	4935	2230
1892	5034	2245
1893	5050	2564
1894	4255	1509

Table S2. Monthly availability of phenological observations in the primary (n_1) and secondary data (n_2). See Fig. 4 for histograms.

Month	n_1	n_2
J	0	0
F	1	0
M	65	36
A	2375	1414
M	20732	11107
J	19838	10451
J	11606	5807
A	6914	2715
S	3802	58
O	3144	1
N	66	0
D	4	0

Table S3. Temporal variations in phenological observations. Variations are shown for the primary and secondary data given as the numbers of sites with available data, observed taxa, stages/substages, and phenological events. See Fig. 5 for plotted data.

Year	Taxa n_1	Taxa n_2	Sites n_1	Sites n_2	Stages n_1	Stages n_2	Events n_1	Events n_2
1876	87	21	9	8	8	6	154	30
1877	93	25	8	9	8	6	162	33
1878	111	36	48	49	12	7	239	48
1879	105	36	40	40	11	7	180	48
1880	98	37	33	31	9	7	158	48
1881	124	36	46	47	11	7	209	48
1882	125	36	50	45	10	7	201	48
1883	158	36	62	60	9	7	242	48
1884	185	36	63	56	12	7	290	48
1885	139	36	56	55	11	7	229	48
1886	305	36	63	56	11	7	398	48
1887	151	32	60	53	11	6	242	36
1888	160	36	57	51	11	7	254	48
1889	116	36	69	67	11	7	208	48
1890	247	29	70	65	11	7	333	36
1891	288	36	66	68	11	7	370	48
1892	123	36	82	73	11	7	200	48
1893	238	36	83	86	9	7	317	48
1894	96	28	70	64	9	7	161	36

Table S4. Distributions of the dates from the primary (n_1) and secondary data (n_2) given as z-scores. See Fig. 6 for histograms.

Z-score	n_1	Z-score	n_2
-17.125	0	-8.875	0
-16.875	1	-8.625	1
-16.625	0	-8.375	1
-16.375	0	-8.125	0
-16.125	0	-7.875	0
-15.875	1	-7.625	0
-15.625	0	-7.375	0
-15.375	0	-7.125	0
-15.125	0	-6.875	0
-14.875	0	-6.625	0
-14.625	0	-6.375	0
-14.375	0	-6.125	0
-14.125	0	-5.875	1
-13.875	0	-5.625	0
-13.625	0	-5.375	1
-13.375	0	-5.125	0
-13.125	0	-4.875	0
-12.875	0	-4.625	0
-12.625	0	-4.375	0
-12.375	0	-4.125	1
-12.125	0	-3.875	3
-11.875	0	-3.625	2
-11.625	0	-3.375	4
-11.375	0	-3.125	6
-11.125	0	-2.875	6
-10.875	0	-2.625	15
-10.625	0	-2.375	27
-10.375	0	-2.125	34
-10.125	0	-1.875	68
-9.875	0	-1.625	130
-9.625	0	-1.375	289
-9.375	0	-1.125	649
-9.125	1	-0.875	1300
-8.875	0	-0.625	2697
-8.625	1	-0.375	4457
-8.375	1	-0.125	6074
-8.125	0	0.125	5949
-7.875	0	0.375	4459
-7.625	1	0.625	2722
-7.375	0	0.875	1358

-7.125	3	1.125	631
-6.875	2	1.375	283
-6.625	5	1.625	152
-6.375	3	1.875	67
-6.125	1	2.125	33
-5.875	4	2.375	29
-5.625	6	2.625	11
-5.375	8	2.875	11
-5.125	5	3.125	4
-4.875	13	3.375	3
-4.625	13	3.625	3
-4.375	10	3.875	1
-4.125	18	4.125	3
-3.875	19	4.375	0
-3.625	24	4.625	1
-3.375	45	4.875	2
-3.125	62	5.125	2
-2.875	66	5.375	1
-2.625	99		
-2.375	122		
-2.125	159		
-1.875	232		
-1.625	348		
-1.375	615		
-1.125	1163		
-0.875	2055		
-0.625	3941		
-0.375	6195		
-0.125	8321		
0.125	8362		
0.375	6735		
0.625	4304		
0.875	2418		
1.125	1282		
1.375	716		
1.625	392		
1.875	237		
2.125	122		
2.375	100		
2.625	57		
2.875	64		
3.125	48		
3.375	39		
3.625	23		
3.875	18		
4.125	12		

4.375	6
4.625	3
4.875	3
5.125	2
5.375	6
5.625	2
5.875	2
6.125	4
6.375	5
6.625	4
6.875	2
7.125	1
7.375	1
7.625	1
7.875	1
8.125	2
8.375	3
8.625	2
8.875	0
9.125	1
9.375	1
9.625	1
9.875	0
10.125	0
10.375	0
10.625	0
10.875	0
11.125	0
11.375	0
11.625	0
11.875	0
12.125	0
12.375	0
12.625	0
12.875	0
13.125	0
13.375	1
13.625	0
13.875	0
14.125	0
14.375	0
14.625	0
14.875	1
15.125	0
